# Exploring meso- and macro-level contextual factors associated with inequalities in program adoption during statewide scale-up of *TransformUs Primary*, a whole-school physical activity intervention

**DOI:** 10.1186/s12966-025-01810-y

**Published:** 2025-08-18

**Authors:** Elly Ganakas, Jo Salmon, Jiani Ma, Karen E. Lamb, Lisa Barnett, Adrian Bauman, Amanda Telford, Nicola D. Ridgers, Anna Timperio, Harriet Koorts

**Affiliations:** 1 School of Exercise and Nutrition Sciences, Institute for Physical Activity and Nutrition (IPAN), Deakin University, Burwood, VIC Australia; 2 Centre for Epidemiology and Biostatistics, Melbourne School of Population and Global Health, University of Melbourne, Melbourne, VIC Australia; 3 School of Health and Social Development, Institute for Physical Activity and Nutrition (IPAN), Deakin University, Burwood, VIC Australia; 4 Sydney School of Public Health, University of Sydney, Sydney, NSW Australia; 5 National School of Education, Australian Catholic University, East Melbourne, VIC Australia; 6 Alliance for Research in Exercise, Nutrition and Activity (ARENA), Allied Health and Human Performance, University of South Australia, Adelaide, South Australia Australia

**Keywords:** Implementation science, Dissemination, Movement, Sedentary behavior, Child, Public health, Health inequities

## Abstract

**Background:**

Contextual influences on program implementation exist across micro (individual), meso (organization), and macro (government/environment) system levels, yet macro factors are less frequently explored in implementation research. This retrospective study explored differences in adoption across meso- and macro-system levels using data from the 2018–2022 state-wide hybrid effectiveness-implementation trial of *TransformUs Primary*, a whole-school physical activity intervention. Aims were to: (1) assess differences in contextual characteristics between adopting and non-adopting schools and implications for equity, and (2) assess associations between macro-level events and dissemination events with program adoption over time.

**Methods:**

Descriptive statistics (number and %) and chi-squared tests were used to assess differences in contextual characteristics between adopting and non-adopting schools (Aim 1). A time-series analysis of daily data was used to explore associations between the number of dissemination events promoting program awareness (e.g., media, newsletters), macro-level policy events (e.g., education department policies), COVID-19-related remote/on-site learning periods, school term dates (i.e., during/outside of school term) and program adoption (i.e., the number of *TransformUs Primary* registrations per day) (Aim 2).

**Results:**

No differences in either school type (i.e., primary, combined, or special) or community level socio-educational advantage between adopting (*n* = 519) and non-adopting schools (*n* = 1,423) were identified. A higher proportion of adopting schools were located in major cities (71.7% vs. 54.5%; chi-square *p* < 0.001) and were government (public sector) schools (80.0% vs. 63.1%; chi-square *p* < 0.001). Time-series analysis results indicated that the likelihood of adopting *TransformUs Primary* decreased from the date of program launch to the end of the scale-up period (IRR 0.999, 95% CI 0.999–1.000; *p* < 0.005). Both school term date (IRR 5.95, 95% CI 4.78–7.41; *p* < 0.001) and dissemination events (IRR 3.30, 95% CI 2.67–4.06; *p* < 0.001) increased the likelihood of adopting *TransformUs Primary.* Results provided little evidence of an association between the number of policy events or COVID-19-related remote and on-site learning periods and adoption.

**Conclusions:**

Select meso- and macro-level factors had an impact on *TransformUs Primary* adoption. Findings inform the need to work with stakeholders in scale-up to prioritize dissemination strategies that have a discernible impact on adoption above others and consider targeted efforts to reach regional/rural and non-government schools.

**Supplementary Information:**

The online version contains supplementary material available at 10.1186/s12966-025-01810-y.

## Background

Physical activity during childhood is important for mental health and wellbeing [1–3], cardiovascular health [3], and the development of cognitive functions and life skills (e.g., attention, goal setting, and decision-making) [4]. Despite these positive health impacts, globally, school-age children are insufficiently active with over 80% not meeting the recommended average of 60 min of moderate- to vigorous-intensity physical activity per day across the week [5, 6]. More so, *health inequalities* (referred to as measurable differences in health between population groups based on socioeconomic characteristics such as ethnicity, place of residence, and socioeconomic status [7]) exist in adherence to physical activity recommendations. For example, lower adherence to physical activity recommendations is apparent among girls [8–10], children living in areas of disadvantage (e.g., country human development index [HDI] level and region) [10, 11], children with lower parental socioeconomic position and among certain ethnic groups [12]. Aside from the implication that some children may miss out on the benefits of physical activity due to these inequalities, such inequalities also contribute to the increased prevalence of otherwise avoidable adverse health outcomes among disadvantaged groups [13]. As these differences in physical activity are systematic, reasonably avoidable, and are produced and reinforced by social and structural factors [14], they are unjust and thus perpetuate health *inequity* (where health inequities are “unfair and avoidable or remedial systematic differences in health” (7p 5) or opportunities to achieve health [15, 16] among population groups).

Disseminating population-level physical activity interventions at-scale can redress inequities by reaching whole populations [17]. Dissemination involves the targeted distribution of information to spread knowledge about an intervention [18] and scale-up involves expanding the reach of an intervention to more people and settings [19]. Schools provide a unique opportunity to reach a large and diverse cohort of students irrespective of parent socioeconomic status and ethnicity, and thus can provide a key setting for achieving equity during scaling-up [20]. Nonetheless, school-based physical activity interventions can risk widening inequities if, for example, they only include behavioral (e.g., individual behavior change or education) rather than structural (e.g., curriculum and environment change) elements [21–23]. To minimize the risk of widening health disparities, international action plans, such as the *Global Action Plan on Physical Activity* [24] and the International Society for Physical Activity and Health (ISPAH) *Eight Investments that Work for Physical Activity* [25], recommended that school-based physical activity interventions incorporate a whole-of-school approach, addressing all aspects of the school environment including students, teachers, parents and school leadership, as well as the broader social, political and physical environments [26].

Whole-of-school physical activity interventions have been scaled-up internationally [27, 28]. However, few have reported on the representativeness of populations reached [28] and unless measured, it cannot be assumed that interventions delivered at-scale reach all populations universally [23]. Evaluating equality in program adoption is therefore a critical first step in ensuring that all students can benefit from scaled-up whole-school physical activity interventions. Adoption refers to the initial decision or action to employ an intervention [29] and is highly-context specific [30, 31]. In this paper we define context to include “a set of characteristics *and circumstances* that consist of active and unique factors that surround the implementation effort” ([32] p110). Key contextual factors that have previously been identified as having an influence on scale-up and adoption of physical activity interventions include those at the broader environmental or macro-level such as political, social, and sector/workforce factors [30], as well as organizational or meso-level factors such as the socioeconomic and geographic setting of the school, number of students, teacher and physical-education teacher turnover, school-team resistance encountered and per-student financial amount received [33].

In implementation science there have been explicit calls for more research exploring how macro-level contextual factors influence implementation outcomes [32, 34]. Yet, despite the recognized importance of these factors, it is still not common practice to describe specific characteristics of adopting and non-adopting schools when reporting on adoption of scaled-up school-based physical activity interventions [28]. Additionally, the influence of macro-level factors on adoption such as political advocacy and support, policies regarding at-risk groups and statewide initiatives and legislation are less well understood [35, 36]. This has resulted in a significant gap in evidence on how contextual factors can impact adoption and which strategies may help mitigate this. Consequently, should scale-up of whole-school physical activity interventions (and public health interventions in general) continue without knowledge of the factors that impact adoption or the characteristics of schools most and/or least likely to adopt and implement programs, there is a risk that existing inequities in access to opportunities for physical activity will be widened when scaling-up [37].

*TransformUs*, a primary school based physical activity and sedentary behavior intervention, is one example of an efficacious whole of school approach [38, 39] that has been scaled-up state-wide across Victoria, Australia [40]. *TransformUs* incorporates pedagogical and environmental strategies to support children’s physical activity across the school day and at home, including: “(i) health lessons incorporating key physical activity/sedentary behavior messages; (ii) active academic lessons; (iii) active breaks; (iv) changes to the school environment; (v) active homework; and (vi) parent newsletters promoting physical activity” (40 p3). Since 2018, *TransformUs* has been implemented at-scale and is available to all primary schools in Victoria, Australia [40].

The scale-up of *TransformUs* to Victorian primary schools (herein referred to as *TransformUs Primary* to distinguish from the secondary-school version of the program) provides a valuable case study to explore meso- and macro-level contextual factors that impact equality in adoption of scaled-up population-level physical activity interventions. Statewide scale-up occurred across a diverse range of school settings and was significantly impacted by the COVID-19 pandemic which resulted in school closures and a pivot to remote learning across Victorian schools throughout 2020 to 2021. Characteristics of schools that adopted *TransformUs Primary* during the first two years of scale-up (Sep 2018 – Sep 2020) have previously been evaluated [41]. Whilst a higher proportion of schools in major cities and government schools adopted *TransformUs Primary*, no differences were apparent based on school type (i.e., primary, special or combined) or school socio-educational advantage [41]. Although providing some information on *TransformUs Primary* from an equity perspective, the data included covered only a relatively short time frame (the first 24 months of scale-up) and did not explore broader political and environmental contextual factors (i.e., COVID-19) or the scale-up strategy, which can influence adoption at-scale [30]. The purpose of this study was to address these knowledge gaps and explore macro-level contextual influences on adoption of *TransformUs Primary* over a greater scale-up timeframe (2018 to 2022), including having an explicit focus on equity implications for wider roll-out of the program nationally.

The aims of this study were twofold. Firstly, to assess differences in school contextual characteristics between adopting and non-adopting schools in Victoria across the full scale-up period of *TransformUs Primary* and secondly, to assess associations between both macro-level events and dissemination events with program adoption over time.

## Methods

### Study design

This study consisted of a retrospective analysis of program registration data from the state-wide hybrid-effectiveness implementation trial of the *TransformUs Primary* program in Victoria, Australia, occurring from September 2018 to December 2022 [40].

### Sample

All school leaders, teachers and education support staff (e.g., teachers’ aides/learning support officers) within Victoria were eligible to participate in *TransformUs Primary* during scale-up [40]. For Aim 1 the sample consisted of all primary (i.e. preparatory to Year 6), combined (i.e., preparatory to Year 12) and special schools (i.e., schools that specialize in teaching students with disability and high needs) in Victoria (*n* = 1,942) listed in the Australian Curriculum Assessment and Reporting Authority (ACARA) *MySchool School Profile 2022* dataset [42]. For Aim 2 the total number of registrations each day during the hybrid effectiveness-implementation trial from 12 September 2018 to 15 December 2022 (i.e., 1,556 days) was considered.

### Data cleaning

Program registration data were first cleaned for duplicate entries and entries containing invalid email addresses. As multiple registrations could be from the same school, once the earliest date of program registration was identified, subsequent entries were excluded from the dataset and registrant names and email addresses removed. This enabled assessment of school registration for analysis rather than individual registration – as setting-level participation was of key interest to this study. The final list of *TransformUs Primary* registrations which spanned the period of 12 September 2018 to 15 December 2022 was used to create two datasets for analysis.

For Aim 1, the MySchool School Profile 2022 dataset [42] was adapted to remove secondary-only schools and schools outside of Victoria. An additional variable was included in which schools were categorized as either having adopted or not adopted *TransformUs Primary* based on registration data (adoption criteria defined below).

**Fig. 1:**
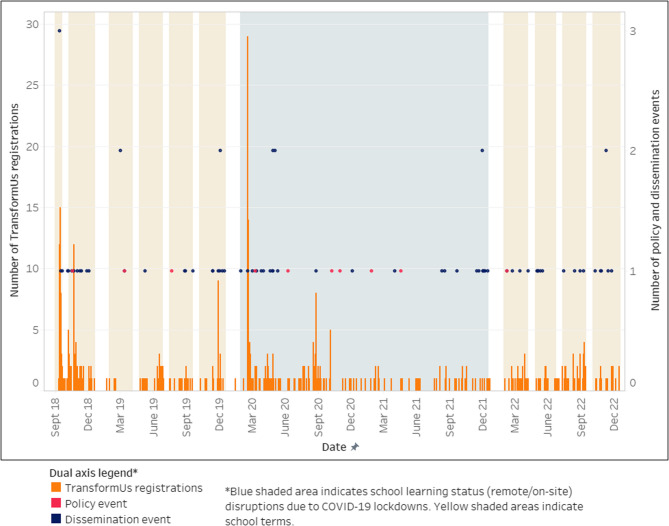
Total setting-level registrations per day, key events and other contextual factors

For Aim 2, a daily time-series dataset was created from the date of website launch (12 September 2018) to the date of the last registration received (15 December 2022) prior to the end of the hybrid effectiveness-implementation trial funding period (31 December 2022). This dataset included the total number of registrations received per day at the setting (school) level, total daily dissemination events, COVID-19-related remote and on-site learning periods for primary school students, policy events, and school term dates (in school term or holidays). This dataset was used to create Fig. 1.

### Measures

#### Outcome variables

For the purpose of this study we defined adoption as a school’s or teacher’s decision to register for *TransformUs Primary* via the *TransformUs* website [41], as this was considered to represent the intention to try the program [29]. From September 2018 to November 2021 the website initially captured program registrations as part of two unique pathways (school or teacher level) with each group exposed to different questions. The website was subsequently updated to amalgamate the forms and conditional logic was used to show participants relevant questions based on their role. Given that this study aimed to assess the influence of contextual factors on adoption at the setting (school) level, each school was only counted once in the analysis irrespective of the number of program registrations and registration types received per school. As such, a school was considered to have adopted *TransformUs Primary* if one or more of the following criteria were met: (i) a school-level registration form had been submitted, (ii) one or more teacher-level registration forms had been submitted, or (iii) one or more amalgamated registration forms had been submitted. Where multiple forms had been submitted per school, the earliest date of registration was selected as the date of adoption. Registration forms were date and time stamped, and the sum of the total number of registrations received for each calendar day was used for the analysis. As registration data was collected via standardized forms with a mandatory response field for school name, registrant name, and email address, registration data relevant for the purpose of this study was complete.

For Aim 1, adoption (i.e., *TransformUs Primary* adopted or not adopted) was the outcome variable analyzed. For Aim 2, the number of schools that adopted *TransformUs Primary* per day (termed ‘program adoption’) was the outcome variable analyzed.

#### Exposure (independent) variables

For Aim 1, exposure variables included school type (primary, combined, special), school sector (government, independent [private sector], Catholic [schools governed by Catholic education proprietors]), location (major cities, inner regional, outer regional, remote, and very remote), and Index of Community Socio-educational Advantage (ICSEA) value as reported in the MySchool School Profile 2022 dataset [42]. In this dataset location is based on the Australian Bureau of Statistics Remoteness Classification, which is a national measure of relative geographic access to services [43]. ICSEA values, which serve as a “way to enable fair and meaningful comparisons between schools” of student performance, are derived from national school and student-level variables (44 p1). School and student-level variables included in ICSEA are remoteness, percent of Indigenous students enrolled, and parental occupation and education level of enrolled students [44]. ICSEA values feature on a scale with a median value of 1000 and standard deviation of 100, ranging from approximately 500 (extremely disadvantaged) to 1300 (extremely advantaged) [44]. In this study ICSEA values were categorized into three groups; low-ICSEA, mid-ICSEA, and high-ICSEA; based on half a standard deviation above and below the sample mean of 1032. As such, 33% of the sample was categorized as low-ICSEA (ICSEA less than 994), 37% as mid-ICSEA (ICSEA between 994 and 1070), and 31% as high-ICSEA (ICSEA above 1070). A similar approach to categorization using the national mean and standard deviation has been adopted in previous literature assessing equity in Australian schools [45, 46].

Exposure variables for Aim 2 included daily data on the number of key dissemination events (coordinated by the research team or strategic partners, detailed below), Victorian primary school student remote or on-site learning status during COVID-19, the occurrence of a government education, health or sport policy event, and school term (i.e., school in session or not). We chose to include learning status during COVID-19 in this analysis as the pandemic was a macro-level ‘critical incident’ [47] that took place in the midst of scale-up, which would be imprudent to disregard given the impact on society at-large and children in particular [48]. In this study, the identification of a policy event was informed by the notion of policy as a “context to understand” and a fixed determinant of implementation outcomes ([49] p3). We utilized the definition of policy as “the explicit (and thus documented) formal decision by an executive agency to solve a certain problem through the deployment of specific resources, and the establishment of specific sets of goals and objectives to be met within a specific time frame” ([50] p51). Considering this, we included government education, health, and sport department policies and initiatives that explicitly referred to *TransformUs Primary*, and the provision of grant funding in association with such policies and initiatives (detailed in Table 1).

The dissemination strategy for the scale-up of *TransformUs Primary* started with an official launch by the Deputy Premier of Victoria and Minister for Education and involved the sharing of program information and updates via email lists, news articles, social media, teacher professional learning networks, and teacher professional development conferences and workshops [40]. Program information was shared by the research team and seventeen community and educational strategic partners [40]. The dissemination variable included in analysis was thus defined as active efforts to spread information about *TransformUs Primary* to target audiences (e.g., schools, teachers, health promotion professionals), by either a member of the research team or a partner organization who supported scale-up. Event categories and descriptions are summarized in Table 1. Individual events are listed in Additional File 1.

Policy events and on-site/remote learning periods during COVID-19 were identified via a search of online grey literature, custom data-requests to government organizations, and in discussions with program stakeholders. A log of key dissemination events was provided to the research team by the *TransformUs* program team (Additional File 1). School term dates were identified via the Victorian Government Department of Education website [51].

**Table 1:** Exposure variable types, categories and descriptions

Type	Category	Description
Dissemination actions and events	Article	Any standalone piece that is published on a partner’s website (e.g., a blog) or released as hard copy specifically featuring a story on *TransformUs Primary*. Different to a newsletter and does not include the media.
	Conference	When a member of the *TransformUs Primary* research team presents at a teacher or professional development conference either held by a partner or external organization.
	Email	Any standalone advertisement about *TransformUs Primary* sent via email.
	Media	Newspaper articles initiated or contributed to by the *TransformUs Primary* team.
	Newsletter	Promotion of *TransformUs Primary* by partners in regularly released content to schools or teachers (e.g., e-bulletin, circular).
	Presentation	Any presentation on *TransformUs Primary* to target audiences that was not an official conference (e.g., seminar, meeting, professional learning workshop) usually by a member of the *TransformUs* research team, but occasionally by strategic partners.
	Website	Link to *TransformUs Primary* content included on partners own website (e.g., video post, *TransformUs* resources made available).
	Other	Dissemination events not captured above (e.g., promotional materials distributed, podcast interview).
Policy actions and events	Funding opportunity	Availability of public grant funding for schools to implement *TransformUs Primary*.
	Resource released	Toolkits and other resources referring to *TransformUs Primary* released.
	Policy released	Policy document (e.g., consensus statement) referring to *TransformUs Primary* released.
COVID-19 learning status	All remote learning	Primary school students in regional/rural and metropolitan Melbourne are required to learn from home.
	Some remote learning	Some primary school students (either regional/rural or metropolitan) are required to learn from home.
	No remote learning	All primary school students are learning on-site at school.
School term dates	School term	Term 1, 2, 3, and 4 dates for Victorian Government schools.
	School holidays	Holiday dates for Victorian Government schools.

### Statistical analysis

For Aim 1, differences in each meso-level school characteristic (school sector, school type, location, ICSEA group) between adopting and non-adopting schools were examined using cross-tabulations of numbers and percentages and Chi-squared tests of association.

To address Aim 2, a time-series analysis was performed to examine changes in the number of schools adopting *TransformUs Primary* over time and to assess whether adoption was influenced by the number of dissemination events, number of policy events, COVID-19-related remote and on-site learning periods, and school term dates from 12 September 2018 to 15 December 2022. Given the study aimed to understand adoption in a real-world context which involves a complex and dynamic interaction of events [52], all exposure variables were included together in the regression model to simultaneously account for factors hypothesized to impact the outcome. As this model utilized aggregated daily data, adoption data for Aim 2 was undifferentiated by school characteristics.

As the outcome variable (i.e., *TransformUs Primary* adoption over time) consisted of rare event count data with a high number of zero values, resulting in overdispersion (i.e., greater variability than anticipated), a negative binomial model was deemed most appropriate as opposed to other time-series models suited to continuous data, such as ARIMA and vector autoregressive models [53, 54]. To account for the presence of a negative trend in the outcome variable and the potential for autocorrelation due to irregularity and seasonality inherent in time-series data [54], we utilized a negative binomial generalized linear model with Newey-West estimators of variance. Newey-West estimators are designed for time-series analysis and assume autocorrelation and heteroskedasticity in the data [55]. Exposure variables were also lagged in various ways to account for potential delays in registration following an event, and sensitivity analyses were conducted to determine the most appropriate lag (see Additional File 1). In the results presented, dissemination events were lagged by one day (i.e., the number of events on the prior day was assumed to directly influence the number of registrations in the subsequent day), and policy events included as a weeklong (i.e., seven-day) period in the model. COVID-19 learning status was modelled as a period of time over consecutive days where each day was assigned a categorical value corresponding to the type of learning (i.e., all students on-site, some on-site, or all students remote), with the reference value representing dates outside of the COVID-related learning disruption period which spanned from 28 January 2020 to 17 December 2021.

To support the interpretation of findings, daily adoption rates were mapped against key events (policy and dissemination) and other contextual factors (COVID-19-related on-site and remote learning periods and school term dates) using a dual-axis bar chart. Summary statistics and a boxplot were generated to interpret the chart and identify days on which an increase in adoption occurred (i.e., outlier values) and the corresponding events.

STATA-SE 18.0 (StataCorp LCC, 2023) was used for all statistical analyses. Tableau Desktop (Salesforce Inc., 2024, California, USA) was used to create Fig. 1.

## Results

### Participating school characteristics

A total of 520 Victorian schools adopted *TransformUs Primary* from 12 September 2018 to 15 December 2022. For Aim 1, 519 adopting schools and 1,423 non-adopting schools were included in the analysis. One school which had adopted *TransformUs Primary* but closed prior to 2022 was excluded as there was no comparable information about non-adopters that were open in 2018 but not in 2022. Due to missing data for ICSEA group, 516 adopting schools and 1,411 non-adopting schools were included in this comparison (Table 2).

School type and ICSEA group appeared similar among adopting and non-adopting schools (Table 2). Most adopting and non-adopting schools were primary (82.5% vs. 80.5%, respectively) and approximately a third of both adopting and non-adopting schools were located within each of low, mid and high ICSEA areas. The proportion of Government schools was higher among adopting schools (80.0%) than non-adopting schools (63.1%) (*p <* 0.001). A higher proportion of adopting schools were from major cities (71.7% vs. 54.5%; *p* < 0.001).

**Table 2:** Characteristics of adopting and non-adopting schools

Measure^^^	Category	Adopting, *n* (%) *N* = 519*	Non-adopting, *n* (%) *N* = 1423*	Chi-square test *p*-value
School sector	Government	415 (80.0%)	898 (63.1%)	< 0.001
	Independent	34 (6.6%)	181 (12.7%)	
	Catholic	70 (13.5%)	344 (24.2%)	
School type	Primary	428 (82.5%)	1146 (80.5%)	0.616
	Combined	60 (11.6%)	186 (13.1%)	
	Special	31 (6.0%)	91 (6.4%)	
Location	Major city	372 (71.7%)	775 (54.5%)	< 0.001
	Inner regional	113 (21.8%)	489 (34.4%)	
	Outer regional	33 (6.4%)	155 (10.9%)	
	Remote	1 (0.2%)	4 (0.3%)	
	Very remote	0 (0.0%)	0 (0.0%)	
ICSEA group	Low ICSEA	175 (33.9%)	451 (32.0%)	0.101
	Mid ICSEA	169 (32.8%)	536 (38.0%)	
	High ICSEA	172 (33.3%)	424 (30.1%)	

^Data obtained from the MySchool School Profile dataset [42]. *516 adopting schools and 1,411 non-adopting used in ICSEA group analysis due to missing data for this characteristic

### Descriptive analysis of adoption over time and associated key events

Figure 1 illustrates adoption over time (i.e., total number of setting level registrations per day) and the number of dissemination and policy events, school terms and the period of COVID-19-related school disruptions.

Adoption occurred on 294 out of the 1,556 days from 12 September 2018 to 15 December 2022. Of these 294 days, the median number of adopting schools per day was 1 (interquartile range [IQR] 1–12) and the maximum number on one day was 29. Excluding days on which no adoption occurred, the box plot for adoption indicated that values of four or more were outliers (see Additional File 1). Outlier values for adoption were recorded on 19 days during the scale-up period, listed in Table 3 along with associated events and other contextual factors. At the start of the scale-up period in September 2018, the largest increases in adoption within one week (*n* = 40) occurred after media releases about the program launch (Table 3). In the middle of the scale-up period in February 2020, a large increase within one week (*n* = 53) followed the release of a Victorian Government Department of Education and Training newsletter. 95% (74 out of 78) of dissemination events occurred during school term (Additional File 1).

**Table 3:** Key dates with an elevated number of registrations received and associated events

Date	Registrations	Associated events and other contextual factors^1,2^
14 Sep 2018 15 Sep 2018 16 Sep 2018 17 Sep 2018	12 15 5 8	**Dissemination event**: Media releases about the launch of *TransformUs Primary* occurred on 13 September 2018 across multiple sources. **Context**: School Term 3
08 Oct 2018	5	**Dissemination event**: *TransformUs Primary* was included in two partner newsletters released on 7 and 8 October. **Context**: School Term 4
23 Oct 2018 24 Oct 2018 29 Oct 2018	12 7 4	**Policy event**: Achievement Program online portal launched on 20 October 2018 which includes TransformUs Primary as a ‘supporting program’ option. **Dissemination event**: A Victorian Government Department of Education and Training newsletter was circulated to government schools promoting *TransformUs Primary* on 23 October 2018. **Context**: School Term 4
28 Nov 2019	9	**Dissemination event**: A member of the *TransformUs Primary* team presented at a national conference from 28 to 29 November 2019. **Context**: School Term 4
19 Feb 2020 20 Feb 2020 21 Feb 2020 23 Feb 2020	29 14 6 4	**Dissemination event**: An article about *TransformUs Primary* was published by the Victorian Government Department of Education and Training on 18 February 2020. **Context**: School Term 1 during COVID-19 period – all students learning on-site.
18 Aug 2020 19 Aug 2020	4 4	No discrete events identified. **Context**: School Term 3 during COVID-19 period – all students learning remotely.
26 Aug 2020 27 Aug 2020	4 8	**Dissemination event**: Members of the *TransformUs* team co-authored a media article published on 26 August 2020, which referred to TransformUs. **Context**: School Term 3 during COVID-19 period – all students learning remotely.
05 Oct 2020	5	No discrete events identified. **Context**: School Term 4 start-date. COVID-19 period – some students learning remotely and some learning on-site.
09 Sep 2022	4	**Dissemination event**: A member of the *TransformUs* team presented to a community sport organization on 8 September 2022. **Context**: School Term 3

^1^Events listed in this table occurred up to one week prior to the spike in registrations. For a complete list of event dates see Additional File 1

^2^For details on COVID-19-related learning periods, see: Wright, A. (2022). *Chronology of primary and secondary school closures in Victoria due to COVID-19* [research note], Parliamentary Library and Information Service, Parliament of Victoria. For details on school term dates, see: https://www.vic.gov.au/school-term-dates-and-holidays-victoria

### Time-series analysis of adoption over time

Results from the time-series analysis fitted using negative binomial regression models (Table 4) indicate an average decrease in the number of registrations by day, although the effect was small (incidence rate ratio [IRR] = 0.999, 95% CI 0.999–1.000, *p* = 0.004). The number of dissemination events was found to be positively related to the number of registrations (IRR = 3.30, 95% CI 2.67–4.06, *p* < 0.001). These results indicate that for every additional dissemination event in the day prior there is a more than three-fold increase in adoption. School term dates appeared to impact the number of registrations. Compared to dates outside of term the estimated adoption was almost six times higher during school terms (IRR = 5.95, 95% CI 4.78–7.41, *p* < 0.001). There was no evidence of a relationship between policy events and adoption (IRR = 1.12, 95% CI 0.66–1.89, *p* = 0.670). Although results suggested increased adoption with school holidays during COVID-19 compared to dates outside of the COVID-related school disruption period (IRR = 1.80, 95% CI 1.09–2.96, *p* = 0.021), there were no apparent differences between the other COVID-19 learning status categories and dates outside of the COVID-related school disruption period (Table 4).

**Table 4:** Time-series analysis of daily number of registrations to *TransformUs primary* by key events*

Model covariate	Incidence rate-ratio (95% CI)	*p* value
Date	0.999 (0.999–1.000)	0.004
Number of dissemination events (lagged by one day)	3.30 (2.67–4.06)	< 0.001
Number of policy events (modelled as a seven-day period)	1.12 (0.66–1.89)	0.670
COVID-19 learning status		
Pre/post COVID-19 school lockdown period	-	-
All students on-site (none remote)	0.74 (0.33–1.63)	0.451
Some students on-site and some remote	0.87 (0.57–1.34)	0.533
No students on-site (all remote)	1.31 (0.87–1.98)	0.202
School holidays during COVID-19	1.80 (1.09–2.96)	0.021
School term		
Outside of school term (holidays)	-	-
In school term	5.95 (4.78–7.41)	< 0.001

*Time-series analysis for the period of 12 September 2018 to 15 December 2022 (1,556 days) using negative binomial regression with Newey-West estimators of variance

## Discussion

Using *TransformUs Primary* program registration data, this study sought to identify whether there were differences in adoption based on school socio-demographic characteristics and to examine the potential impact of dissemination events, policy events, and COVID-19-related school closures on program adoption during statewide scale-up. There was evidence of differences in school sector (i.e., government, independent (private), Catholic) and school location between adopting and non-adopting schools. A higher proportion of adopting schools were government schools and schools located in major cities. There was also evidence indicating that the number of dissemination events increased adoption and that adoption was higher during school terms.

Analysis of the full scale-up period of September 2018 to December 2022 also found no apparent differences in school ICSEA group or school type between adopting and non-adopting schools. These results, alongside the differences detected between school sector and location, mirror the findings of the previous evaluation of the first two years of *TransformUs Primary* [41]. Over the shorter adoption period examined previously, results also indicated that a higher proportion of government schools and schools from major cities were registering for the program. Our descriptive key event analysis (not undertaken in the previous analysis) indicated that a spike in registrations occurred following two key dissemination events (the distribution of a newsletter and publication of a website article promoting *TransformUs Primary*) by the Victorian Government Department of Education^1^ whose primary audience are government schools. This finding may explain the higher proportion of government schools registering for *TransformUs Primary*. The higher proportion of schools in major cities registering for the program may suggest that schools in regional and rural areas faced additional barriers to participating in specific dissemination events (e.g., conferences and seminars held in-person). However, as most program dissemination events throughout the scale-up period were delivered in an online format rather than in-person (e.g., media releases, articles, and newsletters), regional and rural schools had similar chances of being reached by key events.

The over-representation of schools from major cities and government schools may be attributed to other factors beyond reach of dissemination efforts. Independent schools and regional/rural schools may provide more opportunities for children to be physically active or already have a student cohort that is sufficiently active. For example, in regional and remote areas children are more likely to meet both physical activity and sedentary behavior recommendations compared to their metropolitan counterparts [56]. In regional Victoria Independent schools are more likely to provide the mandated amount of physical and sport education compared to government schools [57]. If children exhibit sufficient physical activity participation in these areas/sector, there may be less perceived need by school personnel for schools to adopt physical activity promoting interventions such as *TransformUs Primary.* Should this perception be a contributing factor, this suggests a potential misunderstanding of *TransformUs Primary* as a physical education program, rather than one which seeks to facilitate student engagement in the classroom through movement [58]. Previous research has shown that school leaders’ understanding of the health and academic benefits of *TransformUs Primary*, as well as program alignment with school goals, are key drivers of adoption [59]. As such, ensuring the benefits of *TransformUs Primary* are well communicated and highlighting how active learning increases student engagement and academic outcomes [60] may be pertinent in these contexts.

This study found similar proportions of low, mid and high ICSEA schools among both adopting and non-adopting schools. This may be explained, in part, by the accessible nature of *TransformUs Primary* resources which are available online for free, as opposed to other forms of professional learning for teachers which require face-to-face attendance that incur incidental costs of employing relief teachers to cover absences for training. Additionally, suggestions for low-cost environmental changes are provided on the program’s website which may reduce inequality in adoption. The Department of Education’s Active Schools grant funding, which incentivizes socioeconomically disadvantaged schools to improve physical activity outcomes among students by implementing whole-school physical activity initiatives that include active classrooms, recess and lunchtime breaks, before and after school programs, active travel, and creating a supportive school environment [61] may also have contributed to this lack of difference for adopting schools (*TransformUs* was incorporated into this scheme). Further research exploring stakeholder perspectives on the impact of policy initiatives on socioeconomically disadvantaged schools may help elucidate this finding.

With respect to our second aim exploring the impact of macro-level contextual factors on program adoption, results of the time-series analysis indicated that adoption of *TransformUs Primary* was positively related to the number of dissemination events and school term dates. Dissemination events increased adoption three-fold, while adoption was five-times greater during school terms. As almost all dissemination events occurred during the school term, the timing of dissemination events is likely to have contributed to this finding. Previous qualitative research with partner organization representatives involved in dissemination during scale-up of *TransformUs Primary* supports this conclusion, as the critical importance of timing strategies appropriately was noted by partners [62]. Scale-up guidance also emphasizes utilizing communication strategies for stakeholder engagement to improve dissemination and scale-up success [63, 64]. Our finding of a significant association between dissemination events and program adoption provides evidence of the importance of doing so with a broad range of strategic partners and suggests that utilizing multiple dissemination channels to “maximize program uptake” (a *TransformUs Primary* implementation strategy) (40 p6) is a valuable approach. Literature specific to the scale-up of physical activity interventions has queried whether a hierarchical influence among stakeholders may be a contributing factor to success [30]. Findings from our key event analysis reflect these considerations, as certain types of dissemination events were more successful than others in eliciting *TransformUs Primary* registrations during scale-up. Specifically, information disseminated by government organizations, media releases with wide reach, and conference presentations resulted in an elevated number of program registrations. Only two newsletters circulated by educational partners that mentioned *TransformUs Primary* appeared to have a slight impact (eliciting five setting-level registrations). All other newsletters circulated by stakeholders appeared to have less of an impact on program registration than those circulated by government partners (potentially indicative of a hierarchical influence among stakeholders). As such, further research exploring the significance of partnering with stakeholders of prominence and those with sector-wide reach is recommended. Moreso, while findings confirm that dissemination strategies impact overall program adoption, from an equity perspective, it is critical to seek to identify which strategies work best for whom, and tailor approaches accordingly [65, 66]. Further research exploring the impact of specific dissemination strategies in different communities and how best to implement tailored strategies is recommended.

Although previous research has identified the political and policy environment as influences on program scale-up [30] and adoption [35], we found no evidence of an association between program adoption and the number of policy events. This may be due to differences in how policy environment is defined and quantified. For example, previous research has identified political priorities, government agendas and financial stability as factors of the political environment [30], while our model utilized aggregated policy events undifferentiated by type. While policy events were undifferentiated by type in our model and only a small number of events were included, it is probable that the type of policy event would impact the likelihood of program adoption. For example, the launch of policies associated with grant funding may have a significant impact on adoption as opposed to policies which only recommend the adoption of whole-school physical activity interventions without the provision of material support. Future in-depth case studies exploring the influence of specific policy agendas and events on school adoption are recommended. We also found no evidence that COVID-19-related on-site and remote learning periods had an impact on program adoption compared to dates outside of this period. We speculate that this finding may have been offset by the spike in registrations received following the publication of a media article during COVID-19 lockdowns in August 2020 featuring *TransformUs Primary*, as well as the perceived value among teachers of implementing innovative programs to engage students during lockdown [67, 68]. Additional online resources for *TransformUs Primary* were also provided during the pandemic in the form of a family activity pack and a remote learning hub for teachers that included downloadable lesson plan ideas for lockdown and supporting videos, which may have facilitated continued engagement with the program despite lockdowns.

### Strengths and limitations

Findings of this study hold practical implications for stakeholders in scale-up strategy planning and selection as an association between dissemination events (e.g., information disseminated by government organizations and media releases with wide reach) and program adoption was illustrated. While the research used an Australian case study, this finding is consistent with recommendations in the *Implementation Strategies Applied in Communities (ISAC) Compilation* [69] and is thus applicable to scaling-up interventions in international education contexts. Additionally, findings of this study highlight sub-groups which may require targeted efforts – specifically regional/rural and non-government schools – in future whole-school physical activity intervention scale-up. We speculate that this is not unique to Australia and that our findings are transferable to similar education contexts internationally, as studies on scaled-up school-based interventions in North America (e.g., Action Schools BC [70])! and England (e.g., the Daily Mile [71]) have also illustrated that most adoptees were from metropolitan areas. Further research exploring the reason for this finding and how best to engage schools from these areas/sectors is thus recommended. This recommendation is consistent with the need for more prevention research, in general, among underrepresented priority populations (which in Australia includes regional and rural populations) [72]. An additional key strength of this study was the ability to assess adoption over time during program scale-up, rather than focus on early adoptees.

Limitations included the inability to differentiate by school characteristic in our time-series analysis and need to aggregate dissemination and policy event dates into higher level categories (e.g., policy events included funding related events, the release of consensus statements and resources) due to the small number of event sub-types within each category. While efforts were undertaken to identify as many key macro-level policy or other events which may have occurred during scale-up via a grey literature search, we cannot guarantee that all events were identified. As we required policy events to have explicitly referred to *TransformUs Primary* for inclusion, policy events which did not refer to the program but may have influenced adoption (e.g., broader education policies that emphasise the benefits of active learning) have not been captured in our analysis. Additionally, our focus on setting-level adoption (where schools were represented once based on the earliest registration received), rather than utilizing multiple registrations per school prevented an assessment of the extent of adoption *within* a school or an exploration of the relationship between dissemination strategies that targeted individuals and the individual-level characteristics (e.g., professional role) of adopters. Overall, as there are methodological challenges in attributing causality in ecological studies more broadly [73], to the further the evidence base on the role of macro-level contextual factors we recommend that future case study research identifies ways to attribute specific events, including distinct types of policy and dissemination events, to adoption over time. To improve approaches to scale-up in practice, additional research that explores how best to engage underrepresented schools during scale-up and ensure equality in adoption, including identifying the types of partnerships that may be most effective for success, is also recommended.

## Conclusions

This study illustrates that select meso-level contextual factors, dissemination events during the scale-up period, and timing of school terms may play a role in influencing whole-school physical activity program adoption during statewide scale-up. These findings hold practical implications for scale-up planning. Specifically: (1) the importance of partnering with stakeholders of prominence (e.g., government departments); (2) the importance of timing dissemination events to align with school terms; and (3) the need to better engage regional/rural and non-government sector schools during scale-up.

## Supplementary Information

Supplementary Material 1.

Supplementary Material 2.

## Data Availability

De-identified datasets used and/or analyzed during the current study are available from the corresponding author on reasonable request.

## Abbreviations

ICSEA: Index of Community Socio-educational Advantage
IQR: Interquartile range
IRR: Incidence rate-ratio
CI: Confidence interval
ISAC: Implementation Strategies Applied in Communities

## Footnotes

1 Formerly Victorian Government Department of Education and Training.
