# Supplementary material for: Exploring meso- and macro-level contextual factors associated with inequalities in program adoption during statewide scale-up of TransformUs Primary, a whole-school physical activity intervention
Source: Int J Behav Nutr Phys Act. 2025 Aug 18;22:111. doi: 10.1186/s12966-025-01810-y (PMC12359990; doi:10.1186/s12966-025-01810-y)
Supplement: Supplementary file 1 — Supplementary Material 1. [file 12966_2025_1810_MOESM1_ESM.docx]

# Event types and dates

## Dissemination events

| Date | Event type |
| --- | --- |
| 13/09/2018 | Media |
| 13/09/2018 | Media |
| 13/09/2018 | Media |
| 18/09/2018 | Media |
| 21/09/2018 | Media |
| 07/10/2018* | Newsletter |
| 08/10/2018 | Newsletter |
| 17/10/2018 | Presentation |
| 23/10/2018 | Newsletter |
| 02/11/2018 | Newsletter |
| 09/11/2018 | Newsletter |
| 13/11/2018 | Presentation |
| 29/11/2018 | Conference |
| 04/12/2018 | Article |
| 01/03/2019 | Article |
| 01/03/2019 | Conference |
| 14/03/2019 | Newsletter |
| 10/05/2019 | Newsletter |
| 28/08/2019 | Article |
| 30/08/2019 | Newsletter |
| 19/09/2019 | Media |
| 13/11/2019 | Website |
| 14/11/2019 | Newsletter |
| 28/11/2019 | Conference |
| 03/12/2019 | Newsletter |
| 05/12/2019 | Newsletter |
| 05/12/2019 | Email |
| 10/12/2019 | Newsletter |
| 16/12/2019 | Newsletter |
| 30/01/2020 | Article |
| 18/02/2020 | Article |
| 03/03/2020 | Newsletter |
| 13/03/2020 | Conference |
| 27/03/2020* | Website |
| 01/04/2020* | Email |
| 21/04/2020 | Newsletter |
| 27/04/2020 | Article |
| 28/04/2020 | Newsletter |
| 28/04/2020 | Article |
| 29/04/2020 | Newsletter |
| 04/05/2020 | Email |
| 04/05/2020 | Email |
| 12/05/2020 | Website |
| 26/08/2020 | Media |
| 05/12/2020 | Article |
| 01/04/2021 | Presentation |
| 11/08/2021 | Article |
| 18/08/2021 | Presentation |
| 22/09/2021* | Presentation |
| 16/11/2021 | Presentation |
| 22/11/2021 | Presentation |
| 30/11/2021 | Presentation |
| 30/11/2021 | Email |
| 02/12/2021 | Article |
| 03/12/2021 | Article |
| 07/12/2021 | Presentation |
| 08/12/2021 | Presentation |
| 16/12/2021 | Email |
| 08/02/2022 | Presentation |
| 23/02/2022 | Presentation |
| 15/03/2022 | Presentation |
| 07/04/2022 | Presentation |
| 02/05/2022 | Presentation |
| 03/05/2022 | Email |
| 05/05/2022 | Presentation |
| 11/05/2022 | Presentation |
| 17/05/2022 | Presentation |
| 14/07/2022 | Email |
| 14/08/2022 | Presentation |
| 29/08/2022 | Presentation |
| 08/09/2022 | Presentation |
| 10/10/2022 | Presentation |
| 26/10/2022 | Other |
| 27/10/2022 | Presentation |
| 09/11/2022 | Presentation |
| 09/11/2022 | Other (promotional materials distributed) |
| 15/11/2022 | Presentation |
| 24/11/2022 | Conference |
| *Indicates date outside of school term (school holidays) | |

**Source**: TransformUs program team dissemination monitoring log [internal document].

## Victorian Government school term dates

| Year | Term | Dates |
| --- | --- | --- |
| 2018 | Term 3 | 16 July to 21 September |
|  |  | 8 October to 21 December |
| 2019 | Term 1 | 29 January (students start 30 January) to 5 April |
|  | Term 2 | 23 April to 28 June |
|  | Term 3 | 15 July to 20 September |
|  | Term 4 | 7 October to 20 December |
| 2020 | Term 1 | 28 January (students start 29 January) to 24 March |
|  | Term 2 | 14 April (students start 15 April) to 26 June |
|  | Term 3 | 13 July to 18 September |
|  | Term 4 | 5 October to 18 December |
| 2021 | Term 1 | 27 January (students start 28 January) to 1 April |
|  | Term 2 | 19 April to 25 June |
|  | Term 3 | 12 July to 17 September |
|  | Term 4 | 4 October to 17 December |
| 2022 | Term 1 | 28 January (students start 31 January) to 8 April |
|  | Term 2 | 26 April to 24 June |
|  | Term 3 | 11 July to 16 September |
|  | Term 4 | 3 October to 20 December |

***Source***: Department of Education. (2024, Oct). *School term dates and holidays in Victoria*. Victorian State Government. <https://www.vic.gov.au/school-term-dates-and-holidays-victoria#previous-years-term-dates>

## COVID-19 remote and on-site learning status for primary school students, 2020 – 2021

| Term | Dates | Category |
| --- | --- | --- |
| Term 1 2020 | 28 January to 24 March | No primary students learning remotely / all learning on-site |
|  | 25 March to 13 April | School holidays during COVID-19 |
| Term 2 2020 | 14 April to 25 May | All primary students learning remotely / none learning on-site |
|  | 26 May to 8 June | Some primary students learning remotely / some learning on-site |
|  | 9 June to 26 June | No primary students learning remotely / all learning on-site |
|  | 27 June to 12 July | School holidays during COVID-19 |
| Term 3 2020 | 13 July to 4 August | Some primary students learning remotely / some learning on-site |
|  | 5 August to 18 September | All primary students learning remotely / none learning on-site |
|  | 19 September to 4 October | School holidays during COVID-19 |
| Term 4 2020 | 5 October to 11 October | Some primary students learning remotely / some learning on-site |
|  | 12 October to 18 December | No primary students learning remotely / all learning on-site |
|  | 19 December to 26 January | School holidays during COVID-19 |
| Term 1 2021 | 27 January to 14 February | No primary students learning remotely / all learning on-site |
|  | 15 February to 17 February | All primary students learning remotely / none learning on-site |
|  | 18 February to 1 April | No primary students learning remotely / all learning on-site |
|  | 2 April to 18 April | School holidays during COVID-19 |
| Term 2 2021 | 19 April to 27 May | No primary students learning remotely / all learning on-site |
|  | 28 May to 2 June | All primary students learning remotely / none learning on-site |
|  | 3 June to 10 June | Some primary students learning remotely / some learning on-site |
|  | 11 June to 25 June | No primary students learning remotely / all learning on-site |
|  | 26 June to 11 July | School holidays during COVID-19 |
| Term 3 2021 | 12 July to 15 July | No primary students learning remotely / all learning on-site |
|  | 16 July to 27 July | All primary students learning remotely / none learning on-site |
|  | 28 July to 5 August | No primary students learning remotely / all learning on-site |
|  | 6 August to 8 August | All primary students learning remotely / none learning on-site |
|  | 9 August to 22 August | Some primary students learning remotely / some learning on-site |
|  | 23 August to 9 September | All primary students learning remotely / none learning on-site |
|  | 10 September to 17 September | Some primary students learning remotely / some learning on-site |
|  | 18 September to 3 October | School holidays during COVID-19 |
| Term 4 2021 | 4 October to 21 October | Some primary students learning remotely / some learning on-site |
|  | 22 October to 17 December | No primary students learning remotely / all learning on-site |

***Source:*** Wright, A. (2022). *Chronology of primary and secondary school closures in Victoria due to COVID-19* [research note], Parliamentary Library and Information Service, Parliament of Victoria. <https://apo.org.au/node/321739>

## National (Australia) and state (Victoria) policy events

| Date | Event category | Description |
| --- | --- | --- |
| 20 October 2018 | Resource released | Achievement Program online portal launched. TransformUs is included as a ‘supporting program’ option in the portal. [1] |
| 14 March 2019 | Resource released | Achievement Program and TransformUs Benchmark Alignment Document launched. As part of the launch, the document was made available as a resource in the Achievement Program portal and promoted to health professionals. [1] |
| 23 July 2019 | Policy released | A Healthier Start for Victorians consensus statement released. TransformUs is listed as an example initiative to support schools to increase students’ physical activity and physical literacy. [2, 3] |
| 11 March 2020 | Funding opportunity | Sporting Schools Plus round one grant funding applications open. TransformUs is listed as an approved provider in the grant guidelines. [4] |
| 9 June 2020 | Funding opportunity | Sporting Schools Plus round two grant funding applications open. TransformUs is listed as an approved provider in the grant guidelines. [5] |
| 8 October 2020 | Resource released | Achievement Program Physical Activity and Movement Toolkit launched, which included links to TransformUs against program requirements where relevant. [1] |
| 1 November 2020 | Policy released | Active Schools Initiative announced by the Deputy Premier of Victoria, and TransformUs mentioned in the media release. [6] TransformUs is also highlighted within Joint Ministerial Statement on Physical Activity for Children and Young People as an Active Classroom initiative. [7] |
| 27 January 2021 | Resource released | Active Schools Toolkit available from Term 1, 2021, which includes TransformUs resources. [8, 9] |
| 19 April 2021 | Funding opportunity | 2021 recipients of the Active Schools Grants Scheme announced by the Deputy Premier of Victoria. TransformUs is mentioned in the media release. [10] |
| 7 February 2022 | Funding opportunity | 2022 recipients of the Active Schools Grants Scheme announced. [11] |

***Sources***

[1] Email communications with Cancer Council representative.

[2] Healthy Eating and Active Living Roundtable. (2019). *A Healthier Start for Victorians: A Consensus Statement on Obesity Prevention.* <https://www.vichealth.vic.gov.au/sites/default/files/Obesity-Consensus-Full-Report.pdf>

[3] ACHPER Victoria. (2019). Healthy Eating & Active Living Roundtable: the crucial role of quality HPE. <https://achper.vic.edu.au/achper/public/news/news-items/Obesity-Policy-Coalition--the-crucial-role-of-quality-health-and-physical-education.aspx>

[4] Sport Australia. (2020). Sporting Schools Plus Grant Guidelines. <https://www.sportaus.gov.au/__data/assets/pdf_file/0006/724254/Sporting-Schools-Plus-grant-guidelines.pdf>

[5] Sport Australia. (2020). Sporting Schools Plus Round 2 Grant Guidelines. <https://www.sportaus.gov.au/__data/assets/pdf_file/0010/737083/35875_Sporting-Schools-Round-2_Grant-guidelines.pdf>

[6] Premier of Victoria. (2020). *Getting Kids Moving Again As We Cut Costs For Families.* Victoria State Government. <https://www.premier.vic.gov.au/getting-kids-moving-again-we-cut-costs-families>

[7] Department of Education and Training. (2020). *Active Schools, Active Kids, Active Communities: A joint ministerial statement on physical activity for children and young people,* Victoria State Government.

[8] Prevention and Population Health Group. (2021). *Creating Active Schools across Victoria*. Victoria State Government. <https://prevention.health.vic.gov.au/blog/posts/creating-active-schools-across-victoria>

[9] Department of Education. (2021). *Active schools toolkit.* Victorian State Government. <https://www.education.vic.gov.au/school/teachers/teachingresources/discipline/physed/Pages/activeschoolstoolkit.aspx>

[10] Premier of Victoria. (2021). *Active schools grants to help get kids moving* [media release]. Victoria State Government. <https://www.premier.vic.gov.au/active-schools-grants-help-get-kids-moving>

[11] Premier of Victoria. (2022). *Young Victorians encouraged to get a move on for health* [media release]. Victoria State Government. <https://www.premier.vic.gov.au/site-4/young-victorians-encouraged-get-move-health>

# Figure A: Box plot of daily total TransformUs Primary registrations

*Reference line at *y*=4

# Time-series sensitivity analyses

## Model A – Unadjusted model, no variance estimators

| **Variable** | **Incidence rate ratio (95% CI)** | ***p* value** |
| --- | --- | --- |
| Date | 0.999 (0.999–0.999) | <0.001 |

## Model B – Unadjusted model, with Newey-West estimators

| **Variable** | **Incidence rate ratio (95% CI)** | ***p* value** |
| --- | --- | --- |
| Date | 0.999 (0.999–1.000) | 0.005 |

The addition of a Newey-West estimator to the unadjusted negative binomial generalized linear model (Model B) has reduced the significance of the date variable compared to Model A. This confirms that utilizing a Newey-West estimator in our model is a suitable approach to address the trend component of time-series data.

## Model C – no lag for dissemination events, policy events modelled as a seven-day period

| **Variable** | **Incidence rate ratio (95% CI)** | ***p* value** |
| --- | --- | --- |
| Date | 0.999 (0.999–1.000) | 0.006 |
| Dissemination events | 1.94 (1.60–2.35) | <0.001 |
| Policy events | 1.00 (0.61–1.62) | 0.985 |
| COVID-19 learning status: |  |  |
| Pre/post COVID-19 school lockdown period | - | - |
| All students on-site (none remote) | 0.78 (0.32–1.90) | 0.589 |
| Some students on-site and some remote | 0.82 (0.52–1.29) | 0.394 |
| No students on-site (all remote) | 1.32 (0.83–2.10) | 0.234 |
| School holidays during COVID-19 | 1.85 (1.09–3.13) | 0.023 |
| School term dates: |  |  |
| Outside of school term (holidays) | - | - |
| In school term | 6.44 (5.17–8.02) | <0.001 |

## Model D – one lag for dissemination events, policy events modelled as a seven-day period

| **Variable** | **Incidence rate ratio (95% CI)** | ***p* value** |
| --- | --- | --- |
| Date | 0.999 (0.999–1.000) | 0.004 |
| Dissemination events | 3.30 (2.67–4.06) | 0.000 |
| Policy events | 1.12 (0.66–1.89) | 0.670 |
| COVID-19 learning status: |  |  |
| Pre/post COVID-19 school lockdown period | - | - |
| All students on-site (none remote) | 0.74 (0.33–1.63) | 0.451 |
| Some students on-site and some remote | 0.87 (0.57–1.34) | 0.533 |
| No students on-site (all remote) | 1.31 (0.87–1.98) | 0.202 |
| School holidays during COVID-19 | 1.80 (1.09–2.96) | 0.021 |
| School term dates: |  |  |
| Outside of school term (holidays) | - | - |
| In school term | 5.95 (4.78–7.41) | 0.000 |

## Model E – two lags for dissemination events, policy events modelled as a seven-day period

| **Variable** | **Incidence rate ratio (95% CI)** | ***p* value** |
| --- | --- | --- |
| Date | 0.999 (0.999–1.000) | 0.004 |
| Dissemination events | 1.91 (1.54–2.38) | 0.000 |
| Policy events | 1.11 (0.63–1.97) | 0.710 |
| COVID-19 learning status: |  |  |
| Pre/post COVID-19 school lockdown period | - | - |
| All students on-site (none remote) | 0.76 (0.33–1.77) | 0.524 |
| Some students on-site and some remote | 0.82 (0.53–1.26) | 0.359 |
| No students on-site (all remote) | 1.34 (0.85–2.10) | 0.208 |
| School holidays during COVID-19 | 1.82 (1.10–2.99) | 0.019 |
| School term dates: |  |  |
| Outside of school term (holidays) | - | - |
| In school term | 6.41 (4.93–8.33) | 0.000 |

## Model F – three lags for dissemination events, policy events modelled as a seven-day period

| **Variable** | **Incidence rate ratio (95% CI)** | ***p* value** |
| --- | --- | --- |
| Date | 0.999 (0.999–1.000) | 0.005 |
| Dissemination events | 1.27 (0.93–1.74) | 0.130 |
| Policy events | 1.12 (0.62–2.00) | 0.713 |
| COVID-19 learning status: |  |  |
| Pre/post COVID-19 school lockdown period | - | - |
| All students on-site (none remote) | 0.77 (0.32–1.82) | 0.545 |
| Some students on-site and some remote | 0.80 (0.51–1.23) | 0.301 |
| No students on-site (all remote) | 1.35 (0.85–2.15) | 0.203 |
| School holidays during COVID-19 | 1.83 (1.10–3.06) | 0.021 |
| School term dates: |  |  |
| Outside of school term (holidays) | - | - |
| In school term | 6.53 (4.94–8.63) | 0.000 |

## Model G – four lags for dissemination events, policy events modelled as a seven-day period

| **Variable** | **Incidence rate ratio (95% CI)** | ***p* value** |
| --- | --- | --- |
| Date | 0.999 (0.999–1.000) | 0.006 |
| Dissemination events | 1.37 (1.01–1.86) | 0.041 |
| Policy events | 1.18 (0.61–2.30) | 0.618 |
| COVID-19 learning status: |  |  |
| Pre/post COVID-19 school lockdown period | - | - |
| All students on-site (none remote) | 0.80 (0.34–1.88) | 0.603 |
| Some students on-site and some remote | 0.83 (0.55–1.25) | 0.371 |
| No students on-site (all remote) | 1.42 (0.90–2.23) | 0.131 |
| School holidays during COVID-19 | 1.79 (1.09–2.95) | 0.022 |
| School term dates: |  |  |
| Outside of school term (holidays) | - | - |
| In school term | 6.12 (4.37–8.56) | 0.000 |

## Model H – five lags for dissemination events, policy events modelled as a seven-day period

| **Variable** | **Incidence rate ratio (95% CI)** | ***p* value** |
| --- | --- | --- |
| Date | 0.999 (0.999–1.000) | 0.007 |
| Dissemination events | 1.08 (0.87–1.34) | 0.474 |
| Policy events | 1.19 (0.62–2.28) | 0.599 |
| COVID-19 learning status: |  |  |
| Pre/post COVID-19 school lockdown period | - | - |
| All students on-site (none remote) | 0.79 (0.34–1.85) | 0.590 |
| Some students on-site and some remote | 0.82 (0.55–1.22) | 0.323 |
| No students on-site (all remote) | 1.42 (0.90–2.23) | 0.135 |
| School holidays during COVID-19 | 1.78 (1.08–2.94) | 0.023 |
| School term dates: |  |  |
| Outside of school term (holidays) | - | - |
| In school term | 6.13 (4.44–8.48) | 0.000 |

## Comparison of AIC and BIC values for each adjusted model

| **Variable** | **AIC** | **BIC** |
| --- | --- | --- |
| Model C | 2178.971 | 2227.120 |
| Model D | **2118.709** | **2166.852** |
| Model E | 2166.495 | 2214.633 |
| Model F | 2165.416 | 2213.547 |
| Model G | 2134.821 | 2182.947 |
| Model H | 2128.096 | 2176.216 |

Model D, where dissemination events have been lagged by one day, is the preferred model as this contains the smallest AIC and BIC values.

## Model I – one lag for dissemination events, policy events modelled as a single-day event

| **Variable** | **Incidence rate ratio (95% CI)** | ***p* value** |
| --- | --- | --- |
| Date | 0.999 (0.999–1.000) | 0.005 |
| Dissemination events | 3.29 (2.67–4.06) | 0.000 |
| Policy events | 0.91 (0.49–1.70) | 0.766 |
| COVID-19 learning status: |  |  |
| Pre/post COVID-19 school lockdown period | - | - |
| All students on-site (none remote) | 0.74 (0.33–1.67) | 0.470 |
| Some students on-site and some remote | 0.87 (0.57–1.34) | 0.531 |
| No students on-site (all remote) | 1.30 (0.87–1.95) | 0.207 |
| School holidays during COVID-19 | 1.80 (1.09–2.97) | 0.021 |
| School term dates: |  |  |
| Outside of school term (holidays) | - | - |
| In school term | 6.01 (5.00–7.22) | 0.000 |

## Comparison of AIC and BIC values for models D and I

| **Variable** | **AIC** | **BIC** |
| --- | --- | --- |
| Model D | **2118.709** | **2166.852** |
| Model I | 2118.931 | 2167.075 |

Model D remains the preferred model, with the smallest AIC and BIC values.
