# Supplementary material for: Exploring meso- and macro-level contextual factors associated with inequalities in program adoption during statewide scale-up of TransformUs Primary, a whole-school physical activity intervention
Source: Int J Behav Nutr Phys Act. 2025 Aug 18;22:111. doi: 10.1186/s12966-025-01810-y (PMC12359990; doi:10.1186/s12966-025-01810-y)
Supplement: Supplementary file 2 — Supplementary Material 2. [file 12966_2025_1810_MOESM2_ESM.docx]

STROBE Statement—checklist of items that should be included in reports of observational studies

|  | Item No. | Recommendation | Page  No. | Relevant text from manuscript |
| --- | --- | --- | --- | --- |
| **Title and abstract** | 1 | (*a*) Indicate the study’s design with a commonly used term in the title or the abstract | 2 | “Retrospective study” stated in abstract. |
|  |  | (*b*) Provide in the abstract an informative and balanced summary of what was done and what was found | 2 | Methods (i.e., use of descriptive statistics, chi-squared tests, and a time-series analysis) and results detailed in abstract. |
| Introduction | | | |  |
| Background/rationale | 2 | Explain the scientific background and rationale for the investigation being reported | 3 – 7 | The background section details the benefits of physical activity, the importance of scaling-up interventions to improve physical activity participation, and the gap in evidence on contextual factors that impact scale-up. |
| Objectives | 3 | State specific objectives, including any prespecified hypotheses | 7 | “The aims of this study were twofold. Firstly, to assess differences in school contextual characteristics between adopting and non-adopting schools in Victoria across the full scale-up period of TransformUs Primary, and secondly, to assess associations between both macro-level events and dissemination events with program adoption over time.” |
| Methods | | | |  |
| Study design | 4 | Present key elements of study design early in the paper | 8 | The subheading *study design* is clearly included at the beginning of the methods section. |
| Setting | 5 | Describe the setting, locations, and relevant dates, including periods of recruitment, exposure, follow-up, and data collection | 8 – 9 | Detailed under the *study design* and *outcome variables* subheadings within the methods section. E.g., “This study consisted of a retrospective analysis of program registration data from the state-wide hybrid-effectiveness implementation trial of the TransformUs Primary program in Victoria, Australia, **occurring from September 2018 to December 2022**.” |
| Participants | 6 | (*a*) *Cohort study*—Give the eligibility criteria, and the sources and methods of selection of participants. Describe methods of follow-up  *Case-control study*—Give the eligibility criteria, and the sources and methods of case ascertainment and control selection. Give the rationale for the choice of cases and controls  *Cross-sectional study*—Give the eligibility criteria, and the sources and methods of selection of participants | 8 | Detailed under the *sample* subheading in the methods section. E.g., “all school leaders, teachers and education support staff (e.g., teachers’ aides/learning support officers) within Victoria were eligible to participate in TransformUs Primary during scale-up…” |
|  |  | (*b*) *Cohort study*—For matched studies, give matching criteria and number of exposed and unexposed  *Case-control study*—For matched studies, give matching criteria and the number of controls per case |  | *Not applicable* |
| Variables | 7 | Clearly define all outcomes, exposures, predictors, potential confounders, and effect modifiers. Give diagnostic criteria, if applicable | 9 – 14 | Detailed under the *outcome variables* and *exposure (independent) variables* subheadings within the methods section. |
| Data sources/ measurement | 8* | For each variable of interest, give sources of data and details of methods of assessment (measurement). Describe comparability of assessment methods if there is more than one group | 9 – 14 | Detailed under the *outcome variables* and *exposure (independent) variables* subheadings within the methods section. |
| Bias | 9 | Describe any efforts to address potential sources of bias | 8 | Noted under the *sample* subheading within the methods section (i.e., program roll-out was universally available – all school leaders, teachers and education support staff in Victoria were eligible to participate). |
| Study size | 10 | Explain how the study size was arrived at | 8 | Detailed under the *sample* subheading in the methods section. |

Continued on next page

| Quantitative variables | 11 | Explain how quantitative variables were handled in the analyses. If applicable, describe which groupings were chosen and why | 8 – 14 | Detailed under the *data cleaning*, *outcome variables*, and *exposure (independent) variables* subheadings of the methods section.  E.g., “In this study, ICSEA values were categorized into three groups; low-ICSEA, mid-ICSEA, and high-ICSEA; based on half a standard deviation above and below the sample mean of 1032.” |
| --- | --- | --- | --- | --- |
| Statistical methods | 12 | (*a*) Describe all statistical methods, including those used to control for confounding | 14 – 16 | Detailed under the *statistical analysis* subheading of the methods section. |
|  |  | (*b*) Describe any methods used to examine subgroups and interactions |  | *Not applicable* |
|  |  | (*c*) Explain how missing data were addressed | 10, 16 | Addressed under the *outcome variables* subheading in the methods section: “As registration data was collected via standardized forms with a mandatory response field for school name, registrant name, and email address, registration data relevant for the purpose of this study was complete.”  Addressed under the *participating school characteristics* subheading in the results section: “One school which had adopted TransformUs Primary but closed prior to 2022 was excluded as there was no comparable information about non-adopters that were open in 2018 but not in 2022.” |
|  |  | (*d*) *Cohort study*—If applicable, explain how loss to follow-up was addressed  *Case-control study*—If applicable, explain how matching of cases and controls was addressed  *Cross-sectional study*—If applicable, describe analytical methods taking account of sampling strategy |  | *Not applicable* |
|  |  | (*e*) Describe any sensitivity analyses | 15 | Detailed under the *statistical analysis* subheading in the methods section. E.g., “Exposure variables were also lagged in various ways to account for potential delays in registration following an event, and sensitivity analyses conducted to determine the most appropriate lag (see Additional File 1).” |
| Results | | | | |
| Participants | 13* | (a) Report numbers of individuals at each stage of study—eg numbers potentially eligible, examined for eligibility, confirmed eligible, included in the study, completing follow-up, and analysed | 16, 18 | Numbers for Aim 1 are detailed under the *participating schools characteristics* subheading in the results section. Numbers for Aim 2 are detailed under the *descriptive analysis of adoption over time and associated key events* subheading. |
|  |  | (b) Give reasons for non-participation at each stage |  | *Not applicable* |
|  |  | (c) Consider use of a flow diagram |  | *Not applicable* |
| Descriptive data | 14* | (a) Give characteristics of study participants (eg demographic, clinical, social) and information on exposures and potential confounders | 16 – 17 | Detailed under the *participating schools characteristics* subheading in the results section. |
|  |  | (b) Indicate number of participants with missing data for each variable of interest | 16 | Detailed under the *participating schools characteristics* subheading in the results section. E.g., “Due to missing data for ICSEA group, 516 adopting schools and 1,411 non-adopting schools were included in this comparison.” |
|  |  | (c) *Cohort study*—Summarise follow-up time (eg, average and total amount) |  | *Not applicable* |
| Outcome data | 15* | *Cohort study*—Report numbers of outcome events or summary measures over time |  | *Not applicable* |
|  |  | *Case-control study—*Report numbers in each exposure category, or summary measures of exposure |  | *Not applicable* |
|  |  | *Cross-sectional study—*Report numbers of outcome events or summary measures | 16 – 21 | Detailed in *results* section. |
| Main results | 16 | (*a*) Give unadjusted estimates and, if applicable, confounder-adjusted estimates and their precision (eg, 95% confidence interval). Make clear which confounders were adjusted for and why they were included | 14 – 16, 21 | Unadjusted estimates reported in *Additional File 1,* adjusted estimates and 95% CI reported under the *time-series analysis of adoption over time* subheading in the results section. Rationale for inclusion of variables detailed under the *statistical analysis* subheading of the methods section. |
|  |  | (*b*) Report category boundaries when continuous variables were categorized | 11 | Detailed under the *exposure (independent) variables* subheading in the methods section (e.g., “In this study, ICSEA values were categorized into three groups…). |
|  |  | (*c*) If relevant, consider translating estimates of relative risk into absolute risk for a meaningful time period |  | *Not applicable* |

Continued on next page

| Other analyses | 17 | Report other analyses done—eg analyses of subgroups and interactions, and sensitivity analyses |  | Sensitivity analysis results reported in *Additional File 1.* |
| --- | --- | --- | --- | --- |
| Discussion | | | | |
| Key results | 18 | Summarise key results with reference to study objectives | 21 | Detailed in first paragraph of discussion section. |
| Limitations | 19 | Discuss limitations of the study, taking into account sources of potential bias or imprecision. Discuss both direction and magnitude of any potential bias | 27 – 28 | Detailed under the *strengths and limitations* subheading of the discussion section. |
| Interpretation | 20 | Give a cautious overall interpretation of results considering objectives, limitations, multiplicity of analyses, results from similar studies, and other relevant evidence | 21 – 28 | Interpretation provided in discussion section with reference to other evidence. |
| Generalisability | 21 | Discuss the generalisability (external validity) of the study results | 27 – 28 | Detailed under the *strengths and limitations* subheading of the discussion section. |
| Other information | |  | | |
| Funding | 22 | Give the source of funding and the role of the funders for the present study and, if applicable, for the original study on which the present article is based | 30 | Detailed under the *funding* subheading of the declarations section. |

*Give information separately for cases and controls in case-control studies and, if applicable, for exposed and unexposed groups in cohort and cross-sectional studies.

**Note:** An Explanation and Elaboration article discusses each checklist item and gives methodological background and published examples of transparent reporting. The STROBE checklist is best used in conjunction with this article (freely available on the Web sites of PLoS Medicine at http://www.plosmedicine.org/, Annals of Internal Medicine at http://www.annals.org/, and Epidemiology at http://www.epidem.com/). Information on the STROBE Initiative is available at www.strobe-statement.org.
